# A method to improve the quality of silica nanoparticles (SNPs) over increasing storage durations

**DOI:** 10.1007/s11051-018-4282-7

**Published:** 2018-08-14

**Authors:** Zihan Lu, Huw Owens

**Affiliations:** School of Materials, The University of Manchester, Manchester, M13 9PL UK

**Keywords:** Silica nanoparticles (SNPs), Solvent varying technique (SVT), Centrifuged SNPs (C-SNPs), Original SNPs (O-SNPs), Storage, Linear model

## Abstract

The solvent varying technique (SVT) provides a simple method for the production of uniform batches of silica nanoparticles (SNPs) of a target average diameter. SNPs synthesized using the SVT have been observed to agglomerate over increasing storage times leading to an increase in average particle diameter. Since the particle diameters of the SNPs produced using the SVT may vary over increasing storage durations, the previous model, suggested by Gao et al., which is based on the diameter of the original SNPs, is unreliable when predicting a target particle diameter using the initial volume of ethanol. A centrifuge and replacement of solvent method has been applied in this investigation to the SNP solutions created using the SV technique. This reduces the amount of unused reactants in the centrifuged colloidal suspensions, which further improves the quality of the SNPs and hence any subsequent photonic crystals. Post centrifuge and replace, the morphology of the centrifuged particles is more uniform than that of the original particles, which has been evaluated using SEM micrographs. The face-centered cubic (FCC) structures observed on the surface of the photonic crystal films have also been imaged using a SEM. A linear equation for the prediction of the SNP diameters for a given initial amount of ethanol is proposed based on the centrifuged SNP diameters. The particle diameter measurements for the new equation were recorded using a DLS instrument. The dispersion of the SNPs was also recorded using DLS. The morphology of the surface of the particles has been confirmed using TEM micrographs.

**Electronic supplementary material:**

The online version of this article (10.1007/s11051-018-4282-7) contains supplementary material, which is available to authorized users.

**Graphical abstract:**
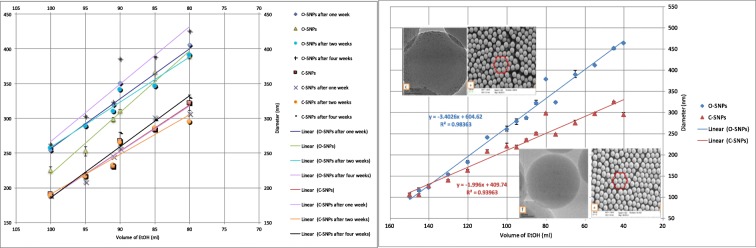
ᅟ

## Introduction

Silica nanoparticles (SNPs) are commonly used to coat materials in order to produce structural colors as well as other beneficial surface modification effects (Gaillou et al. 2008). Structural colors have unique features compared with traditional dyes (Baumberg and Snoswell 2009), which makes producing artificial structural colors desirable (Braun 2011; Fudouzi and Xia 2003). Colloidal crystallization (Gao et al. 2016a, b; van Blaaderen, Ruel and Wiltzius 1997; Park, Qin and Xia 1998; Lee et al. 2013; Pieranski 1983; Xia et al. 2000) is a frequently applied method when producing artificial structural colors. Synthesizing silica nanoparticles (SNPs) is an established method (Santamaría Razo et al. 2008) for the production of structural color. There are several standard published methods that outline the production of silica nanoparticles, which are illustrated in the disquisition of Tiler in 1979 (Iler and Iler 1979). The most popular method uses four chemicals: ammonia, distilled water, ethanol, and tetraethyl orthosilicate (TEOS) to synthesize the SNPs (Stöber, Fink and Bohn 1968; Giesche 1994). Ammonia is used as the catalyst, with the hydrolysis and condensation occurring between the tetraethyl orthosilicate (TEOS) and distilled water (Gao et al. 2016a, b; Stöber, Fink and Bohn 1968; Giesche 1994; Gao 2016). In 1956, Kolbe discovered the chemical reaction theory underpinning this method. Since his discovery, substantial research has been focused on this area (Kolbe 1956). In 1968, the study of this reaction system had a breakthrough (Stöber, Fink and Bohn 1968). Stöber, Fink, and Bohn proposed a chemical technique to control the particle size of the silica which was in the micron size range (Stöber, Fink and Bohn 1968; Galisteo-López et al. 2011). This technique is now referred to as the Stöber or SFB method (Stöber, Fink and Bohn 1968). Although the SNPs can be efficiently synthesized using the Stӧber method, the limitation of this method is that the precise prediction of final particle diameter is difficult to achieve (Gao et al. 2016a, b; Galisteo-López et al. 2011). On the basis of the Stӧber method, several researchers (Iler and Iler 1979; Gao 2016) have published experiments to attempt to predict the diameter of the final particles (Gao 2016). However, the difference between the predicted diameter and the experimental diameter still shows considerable variation (Gao 2016), and their methods cannot be applied to accurately predict the final particle size. In 2016, based on the Stӧber method, Gao et al. proposed the solvent varying technique (SVT) and provided a supporting equation that allowed the user to produce a batch of SNPs with a particular target diameter by only varying the initial amount of ethanol in the solution (Gao et al. 2016a, b, 2017a, b). Gao et al. synthesized uniform SNPs by mixing 8 ml ammonia hydroxide, 3 ml distilled water, and a certain calculated initial volume of ethanol (Gao et al. 2016a, b; Gao 2016). Once the solution was heated to a temperature of 60 °C, 6 ml TEOS was added into the mixture. Using the SVT, SNPs with target diameters of between 207 and 350 nm have been prepared and these particles can be used to produce tunable structural colors by a process of natural gravity sedimentation (Gao et al. 2016a, b).

One limitation of the synthesis technique and equation (Gao et al. 2016a, b; Gao 2016) proposed by Gao et al. is that the average particle diameters may be unstable with increasing storage durations. In order to solve this problem, a centrifuge and solvent replacement method has been applied to remove unused chemicals and other impurities in the colloidal suspension and hence improve the stability of the particle diameters. In this paper, a comparison of the morphology and particle diameters of the original and centrifuged SNPs is provided using SEM micrographs of the photonic crystals and DLS measurements of the SNPs in solution. TEM micrographs were used to observe the surface of the original and centrifuged SNPs. The differences between these particles are shown in the TEM micrographs. Based on the diameters of the centrifuged SNPs, a supporting linear equation relating the initial volume of ethanol to the final particle diameter has been suggested.

## Experimental

### Materials

Tetraethyl orthosilicate (TEOS, 99.0%, Fisher Scientific Co., Ltd., UK); ethanol for batch 1 (EtOH, 99.96%, VWR Science Co., Ltd.); ethanol for batch 2 (EtOH, 99.97%, VWR Science Co., Ltd.); ethanol for batch 3 (EtOH, 99.99%, Fisher Scientific Co., Ltd., UK); ethanol for batch 4 (EtOH from batch 3 stored for over 1 month; the concentration of EtOH slightly changes due to storage duration); ammonia hydroxide (NH_3_·H_2_O, 25%, Fisher Scientific Co., Ltd., UK); distilled water (received from USF-ELGA water purifier in the laboratory); All reagents were applied as received without any other purification.

### Synthesis of the silica nanoparticles using the SV technique

Uniform batches of silica nanoparticles (SNPs) of a target diameter were produced by applying the solvent varying technique (SVT). SNP diameters ranged between approximately 100 and 460 nm. The synthesis of the silica nanoparticles was carried out in a single 250 ml round-bottom flask through two reactions, i.e., hydrolysis and condensation polymerization. Firstly, a solution was prepared by mixing 8 ml ammonia hydroxide, 3 ml distilled water, and a certain calculated initial volume of ethanol in the 250-ml flask. The flask was submerged in the water bath. The water bath was placed on a hotplate. Magnetic stirrers were placed both in the flask and the water bath. When the reaction temperature reached 60 °C, the TEOS (6 ml) was added into the flask. The flask was sealed with silicone grease to maintain the temperature through the experiment. Stirring was also maintained during this procedure. It was important to keep the whole reaction temperature at 60 °C and the final mixture was stirred for at least 3 h. After 3 h, the final solution was removed from the flask and transferred into a clean glass bottle.

### Processing of the silica nanoparticles

The samples were centrifuged (Thermo Scientific Sorvall Legend ×1 Centrifuge) twice with the speed of the centrifuge set at 14500 r/s. SNP sample solutions were centrifuged for 10 min. Firstly, the original colloidal suspensions were added into glassware tubes suitable for the centrifuge and then the tubes were placed into the centrifuge. Once centrifuged, the silica nanoparticles collected at the bottom of the tube and the supernatant was transparent. The supernatant was removed and replaced with 25 ml of EtOH. The tubes were then placed into an ultrasonic machine for 30 min to make sure the silica nanoparticles were adequately dispersed in the EtOH. The process was repeated for a second time. On completion of the second centrifuge, the supernatant was removed and the pure silica nanoparticles were left in the tubes. Finally, the EtOH was added into the tubes to disperse the pure silica nanoparticles with the application of the ultrasonic machine.

### Characterization

In this work, the analysis of the diameters of the SNPs was based on the values measured using a dynamic light scattering (DLS, Malvern Zetasizer Nano S) machine, the micrographs from a scanning electron microscope (SEM, Zeiss Ultra 55), and the micrographs from a transmission electron microscope (TEM, CM20). The average hydrodynamic diameter of particles and their polydispersity index (PDI) were measured using dynamic light scattering (Malvern Zetasizer Nano S). The morphology of the SNPs was observed using SEM (Zeiss Ultra 55) and TEM (CM20) micrographs. The particle diameters observed in the micrographs were measured using the ImageJ software package.

## Results and discussions

### A comparison between the photonic crystals produced using the original and centrifuged SNPs

**Fig. 1:**
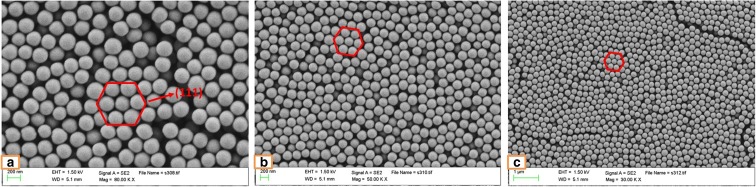
SEM images. Original SNPs produced using 75 ml batch 1 EtOH on silicon wafers (green appearance). Magnification: **a** × 80,000, **b** × 50,000, **c** × 30,000

**Fig. 2:**
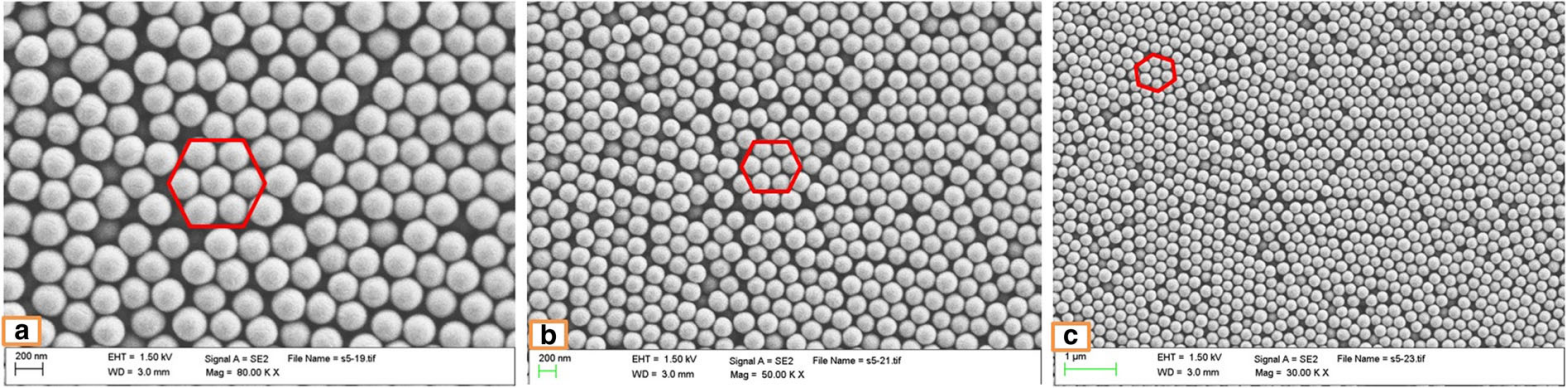
SEM images. Centrifuged SNPs produced using 75 ml batch 1 EtOH on silicon wafers (green appearance). Magnification: **a** × 80,000, **b** × 50,000, **c** × 30,000

Figure 1 shows the morphology of PCs produced from the original SNPs on silicon wafers at three different magnifications. It indicates that the SNPs are arranged in several regular faced-centered cubic (FCC) structures in the photonic crystal (CC) films. The hexagonal structures, shown as the red markers, in the images are in respect of the (111) plane of FCC structure, and this hexagonal close-packed (HCP) arrangement can lead to the CC films having the same optical properties and refractive index in all directions. In addition, Fig. 2 illustrates the SEM images of centrifuged SNPs. There are also several hexagonal structures indicated in the samples, and it can be observed that the centrifuged SNPs form more consistent hexagonal structures in a greater frequency than the original SNPs. The particle diameters can also be calculated using the software package ImageJ. Taking Fig. 1a and Fig. 2a as an example, the original particle diameter is approximately 240 nm while the centrifuged particle size is approximately 200 nm. The SNPs processed using the centrifugation and solvent replacement method have reduced particle diameters when compared to the original particles created using the SV method, over a range of 40 to 130 ml of initial ethanol. In addition to the data showing the changes of particle size, the micrographs can be used to compare the particle density between Fig. 1b and Fig. 2b. It can be observed that, under the same magnification (× 50,000), the amount of centrifuged SNPs is greater than the amount of original SNPs. This is also evident from the DLS data. The difference in particle size may be caused by unused reactants and the centrifuge and solvent replacement method may reduce the impact of these impurities. A more consistent particle diameter leads to the photonic crystals produced from the centrifuged SNPs being more uniform.

**Fig. 3:**
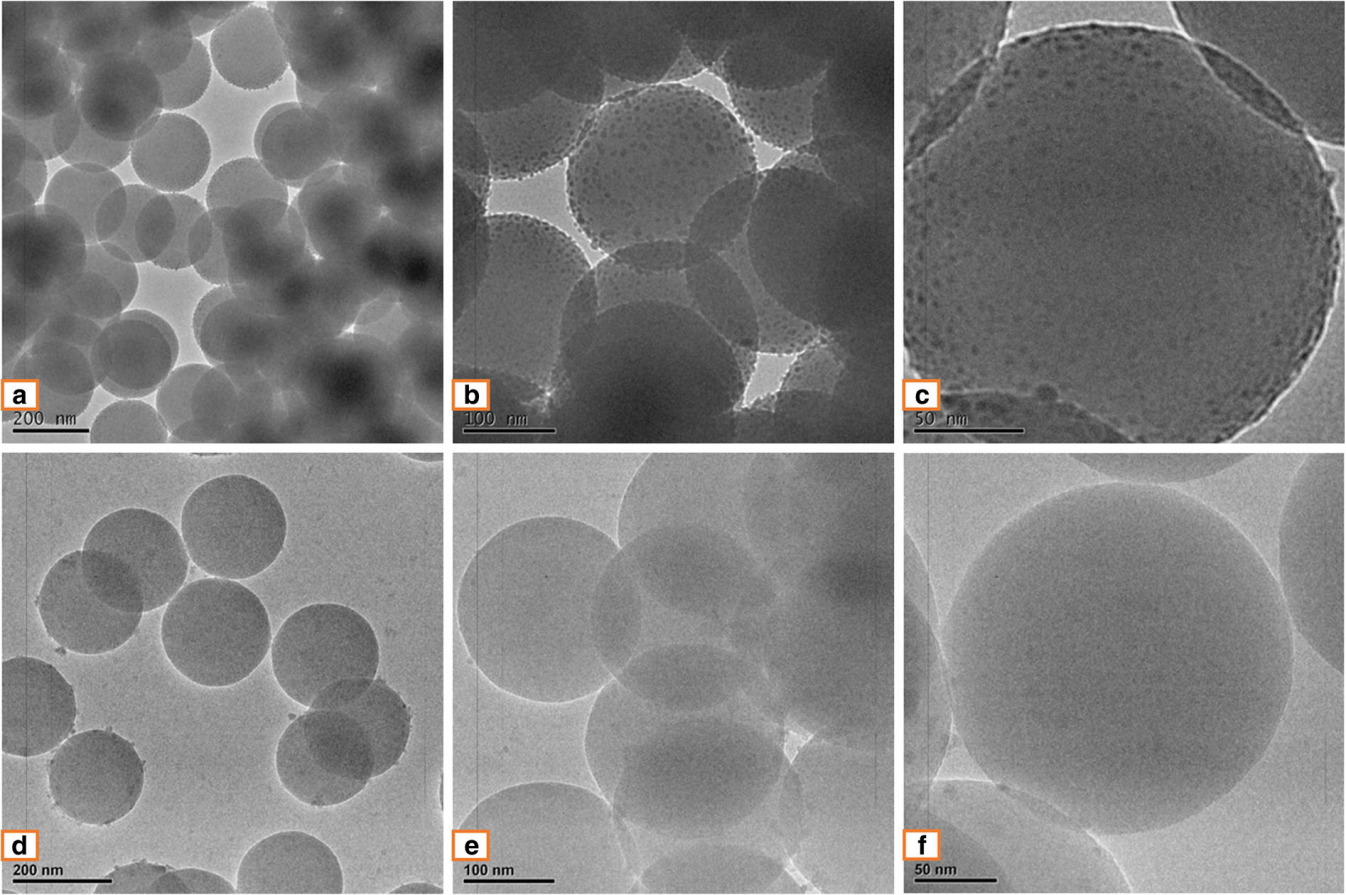
TEM images of SNPs made using 70 ml batch 2 ethanol. Magnification **a**, **d** × 10,000; **b**, **e** × 15,000; **c**, **f** × 27,500. **a**, **b**, and **c** are images of original SNPs, and **d**, **e**, and **f** are images of centrifuged SNPs

In order to demonstrate that the centrifuge and solvent replacement technique can reduce the effect of impurities on SNPs, in this work, micrographs from a TEM have been used to record the morphology of the particles. In Fig. 3a, b, and c., the TEM images of the original SNPs, it can be seen that there are several impurities on the surface of the particles and these impurities may affect the final particle diameters. Figure 3d, e, and f shows the morphology of the particles after applying the centrifuge and replacement method, section “Processing of the Silica Nanoparticles”. Making a visual comparison between the original particles and the centrifuged particles shows that after centrifuging, the SNPs several impurities are removed, Fig. 3c, f. Since the impurities have been removed from original colloidal suspensions, the measurement of the diameter of the centrifuged particles has also been reduced. In Fig. 3, according to the scale bar, the average diameter of the original SNP is 246.7 nm while the average diameter of the purified particle is approximately 221.1 nm. These differences between the particle diameters also cause different colors to be observed.

### Particle diameter changes with increasing storage duration

**Fig. 4:**
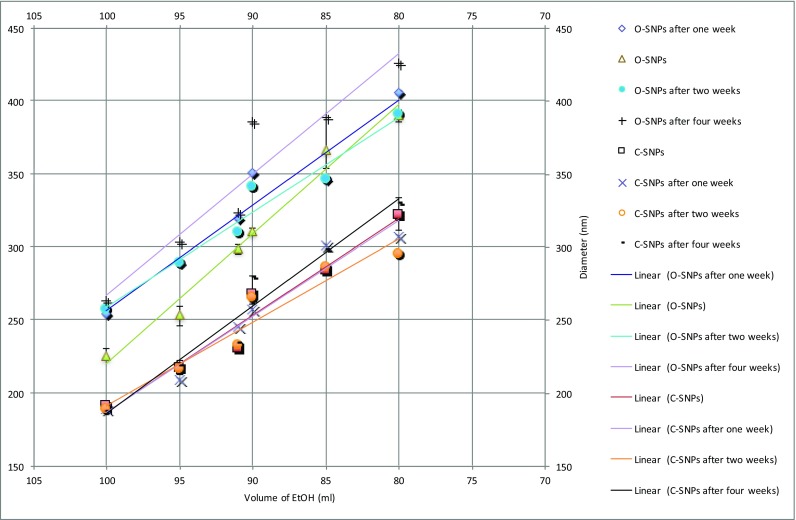
DLS average diameter measurements of the original and centrifuged SNPs (batch 3 ethanol) sampled after 1, 2, and 4 weeks. Error bars show the maximum and minimum DLS diameter measurements

The diameter of SNPs may change with increasing storage durations. The change of particle diameters from the original SVT recipes and the variation of centrifuged particle diameters over 4 weeks, as measured using a DLS instrument, are shown in Fig. 4. It can be seen that the diameter of the original SNPs is generally larger than that of centrifuged particles. From Table 1, it can be seen that for the original particles, as the storage durations become longer, the change of particle size also becomes larger. For instance, the biggest changes of particle diameter for the original SNPs produced using 90 ml of ethanol was approximately 346.88 ± 26.88 nm, while the smallest changes are caused by the particles created by 91 ml of ethanol, 312.86 ± 9.51 nm. This phenomenon suggests that the original particle diameters may be unstable during storage. However, the diameters of the centrifuged SNPs change very little over this time period. For centrifuged SNPs, the biggest changes to the particle diameters are the SNPs which were produced using 80 ml of ethanol, 313.21 ± 13.31 nm, and the smallest observed changes to the particle diameters were shown by particles produced by using 100 ml of initial ethanol, 188.73 ± 1.33 nm. However, such size fluctuations between the sizes of the centrifuged particles are negligible compared to the changes in the original particle diameters, Table 1. Therefore, the centrifuged particles retain a similar diameter over the duration of the study when compared to that of the original SNPs.

**Table 1:** Average diameters of the original SNPs and the centrifuged SNPs over a duration of 4 weeks

	Average diameters and standard deviations of the SNPs (nm)	
Volume of ethanol (ml)	O-SNPs	C-SNPs
100	249.78 ± 14.72	188.73 ± 1.33
95	283.52 ± 18.35	214.78 ± 3.80
91	312.86 ± 9.51	235.44 ± 5.62
90	346.88 ± 26.88	267.19 ± 7.90
85	361.66 ± 17.54	292.04 ± 7.39
80	402.68 ± 14.42	313.21 ± 13.31

### A linear equation for predicting the target diameters of centrifuged SNPs

**Fig. 5:**
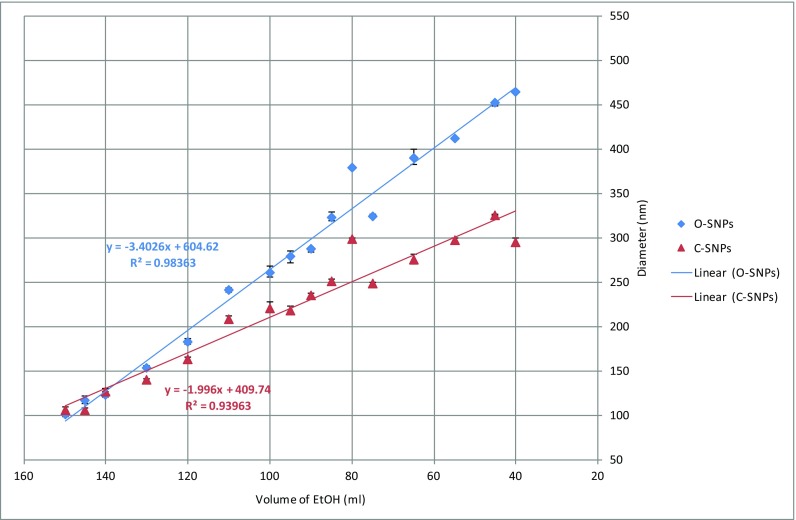
Suggested linear models calculated using the average diameters of the original (blue markers) and centrifuged (red markers) particles (batch 4 ethanol and measured within 1 week of creation). Error bars show the maximum and minimum DLS diameter measurements

The diameters of the centrifuged particles show little variation compared to the diameters of the original particles. A linear equation has been proposed using the DLS data of the average centrifuged particle diameter. As the centrifuged particles show less variation in particle diameter, the model may be more reliable than that suggested by Gao et al. which was based on the DLS average diameter data of the original SNPs (Gao et al. 2016a, b). Sixteen colloidal solutions were produced and the resulting average particle diameters are shown in Table 2. From Table 2, it can be seen that the average diameters of both the original and the centrifuged SNPs are uniform with PDIs less than 0.1. The original particles range in an average diameter between 100.58 and 463.6 nm. They were synthesized using recipes produced from the solvent varying technique (SVT). However, after using the centrifuge and replacement of solvent method, the particle diameters range between 105.93 to 325.4 nm. The differences between the original and the centrifuged particle diameters decrease as the amount of ethanol increases, which suggests that too little ethanol results in the absence of sufficient ethanol to react with the TEOS. This leads to the unreacted TEOS and other impurities remaining in the solution. The results from Table 2 are plotted in Fig. 5 and two linear trend lines have been fitted to the data.

**Table 2:** DLS average particle diameter and PDI results of the original and centrifuged particles

Number	EtOH volume (ml)	Original SNPs (nm)	PDI	Centrifuged SNPs (nm)	PDI
1	40	463.60	0.041	294.53	0.027
2	45	451.57	0.047	325.40	0.044
3	55	411.13	0.042	296.97	0.029
4	65	389.93	0.015	275.00	0.029
5	75	323.90	0.042	248.07	0.030
6	80	378.43	0.042	297.83	0.010
7	85	323.30	0.040	251.20	0.028
8	90	286.83	0.028	235.13	0.004
9	95	278.33	0.036	218.03	0.041
10	100	260.40	0.029	219.87	0.019
11	110	240.83	0.018	208.17	0.015
12	120	182.97	0.017	163.00	0.025
13	130	153.60	0.014	139.40	0.034
14	140	123.30	0.024	127.13	0.061
15	145	116.20	0.034	105.93	0.050
16	150	100.58	0.025	106.27	0.069

Figure 5 shows that there is a negative relationship between the initial volume of ethanol and average particle diameters in the range of initial ethanol applied. Figure 5 shows that the differences between the average diameters of the original particles and that of the centrifuged particles are greater than approximately 20 nm when the volume of ethanol ranges between 40 and 120 ml. However, the distance between the two curves representing the average particle diameter with initial volumes of ethanol between 120 and 150 ml is less than 15 nm. In Fig. 5, the overall gradient of the trend line of the centrifuged SNPs is relatively flat when compared to the gradient of the original SNPs. Therefore, it may be suggested that the residual TEOS and impurities in the colloidal suspensions have a significant effect on the final diameter of SNPs. However, the residual TEOS and other impurities in the colloidal suspensions after centrifugation have been significantly reduced and the particle diameters show less variation. Combined with the data outlined in the section “Particle Diameter Changes with Increasing Storage Duration,” the silica particles can be stored for greater durations after centrifugation with only small changes in average SNP diameter. It can be concluded that the centrifuged particles can be used to create a new and more reliable model to predict target particle diameters based on the initial volume of ethanol. A linear equation is suggested, Eq. 1, where *x* represents the initial volume of ethanol and *y* represents the predicted diameter of the SNPs. *R*^2^ is the coefficient of determination. In this work, it can be seen that the *R*^2^ values of the two equations are both close to 1 (*R*^2^ = 0.98 (original SNPs) and *R*^2^ = 0.94 (centrifuged SNPs)), so the negative relationship between the initial volume of ethanol and particle diameter is confirmed in the new data fits.

1 $$ y=-1.996x+409.74\kern1.5em {R}^2=0.98 $$

2 $$ y=\hbox{-} 3.4026x+604.62\kern0.5em {R}^2=0.94 $$

Equation 2 uses a linear equation to describe the relationship between the average diameters of the original SNPs and the volume of initial ethanol in this experimental range.

## Conclusions

Although the solvent varying technique provides an easy method for producing uniform batches of SNPs of a target diameter, the recipes may not be optimum and leave unused reactants, which may influence the SNP diameters over time. A centrifuge and replacement of solvent technique has been suggested to effectively improve the changes in SNP particle diameter over increased time durations. Centrifuged SNPs exhibit stable particle diameters which are smaller than those produced by the SV method when measured by DLS. The arrangement of the centrifuged SNPs into photonic crystals is more homogeneous and the impurities on the surface of SNPs are also reduced. The reduction of impurities allows the SNPs to be stored for greater durations without significant changes in sphere diameter. Since the centrifuged SNPs have several advantages, a linear model has been established to provide a prediction of a target particle diameter. Two new linear models are proposed to predict the average particle diameter for the SV technique and the centrifuge and replace processed particles. These equations provide a convenient method for researchers to predict target particle diameter according to the initial volume of ethanol.

## Electronic supplementary material

ESM 1 (DOCX 96 kb)
