# Supplementary material for: A method to improve the quality of silica nanoparticles (SNPs) over increasing storage durations
Source: J Nanopart Res. 2018 Aug 14;20(8):213. doi: 10.1007/s11051-018-4282-7 (PMC6096886; doi:10.1007/s11051-018-4282-7)
Supplement: Supplementary file 1 — (DOCX 96 kb) [file 11051_2018_4282_MOESM1_ESM.docx]

# Supporting Information

# **A Method to Improve the Quality of Silica Nanoparticles (SNPs) over Increasing Storage Durations**

School of Materials, The University of Manchester, Manchester, M13 9PL, UK

Corresponding authors E-mail: [Huw.Owens@manchester.ac.uk](mailto:Huw.Owens@manchester.ac.uk)

**Table S1 Average diameters of the original and centrifuged SNPs (Batch 3 ethanol) over one month (DLS results)**

|  | **Diameter of SNP (nm)** | |
| --- | --- | --- |
| **Volume of ethanol (ml)** | **O-SNPs** | **C-SNPs** |
| **100** | 224.87 | 190.90 |
| **95** | 253.43 | 216.50 |
| **91** | 298.83 | 230.33 |
| **90** | 310.30 | 267.60 |
| **85** | 366.07 | 283.93 |
| **80** | 389.80 | 321.43 |
|  | **O-SNPs after one week** | **C-SNPs after one week** |
| **100** | 254.13 | 188.40 |
| **95** | 288.53 | 208.40 |
| **91** | 319.53 | 244.90 |
| **90** | 350.40 | 257.00 |
| **85** | 346.30 | 300.63 |
| **80** | 405.27 | 306.97 |
|  | **O-SNPs after two weeks** | **C-SNPs after two weeks** |
| **100** | 257.30 | 188.30 |
| **95** | 288.87 | 215.80 |
| **91** | 309.70 | 232.47 |
| **90** | 341.10 | 265.10 |
| **85** | 345.73 | 285.53 |
| **80** | 390.40 | 294.90 |
|  | **O-SNPs after four weeks** | **C-SNPs after four weeks** |
| **100** | 262.83 | 187.30 |
| **95** | 303.23 | 218.40 |
| **91** | 323.37 | 234.07 |
| **90** | 385.70 | 279.07 |
| **85** | 388.53 | 298.07 |
| **80** | 425.23 | 329.53 |
